# Cancer cells’ chamber of secrets: the link between micronuclei, chromothripsis and malignancy

**DOI:** 10.1098/rsob.240388

**Published:** 2025-05-14

**Authors:** Truman Raleigh Mcneil, Sweta Sikder, Yamini Dalal

**Affiliations:** ^1^ Center for Cancer Research, National Cancer Institute, NIH, Bethesda, MD, USA

**Keywords:** micronuclei, chromothripsis, cancer, chromosomal instability, mitotic defects, tumorigenesis

## Abstract

Micronuclei exhibit defective proteomes rendering their chromatin vulnerable to fragmentation. This fragmentation process, known as chromothripsis, promotes tumorigenesis by catalysing the activation of oncogenes and the silencing of tumor suppressors. With this role in mind, micronuclei serve as promising targets for therapeutic intervention. This review will explore recent discoveries regarding how micronuclei form, their function in catalysing chromothripsis and how chromothripsis provides a selective advantage for cancer cells.

**Figure:**
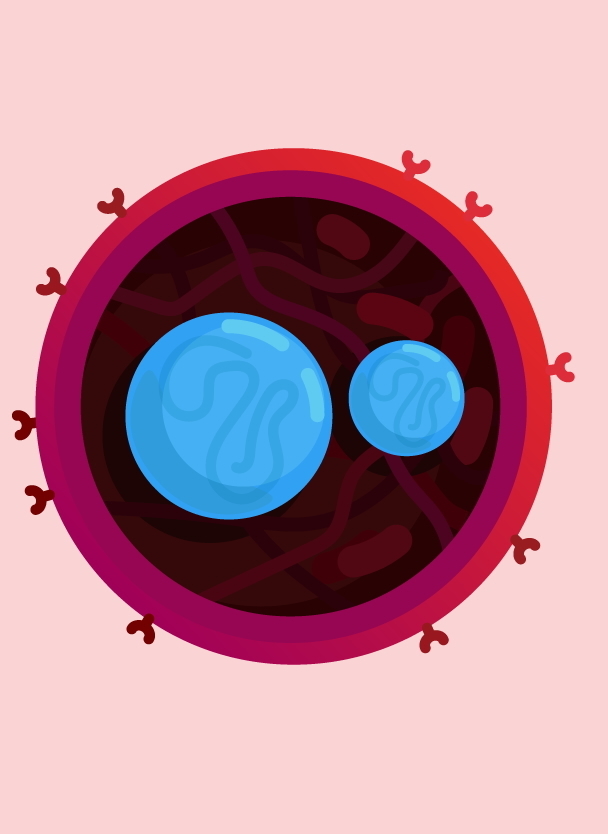

## 1. Introduction

**Figure 1.**
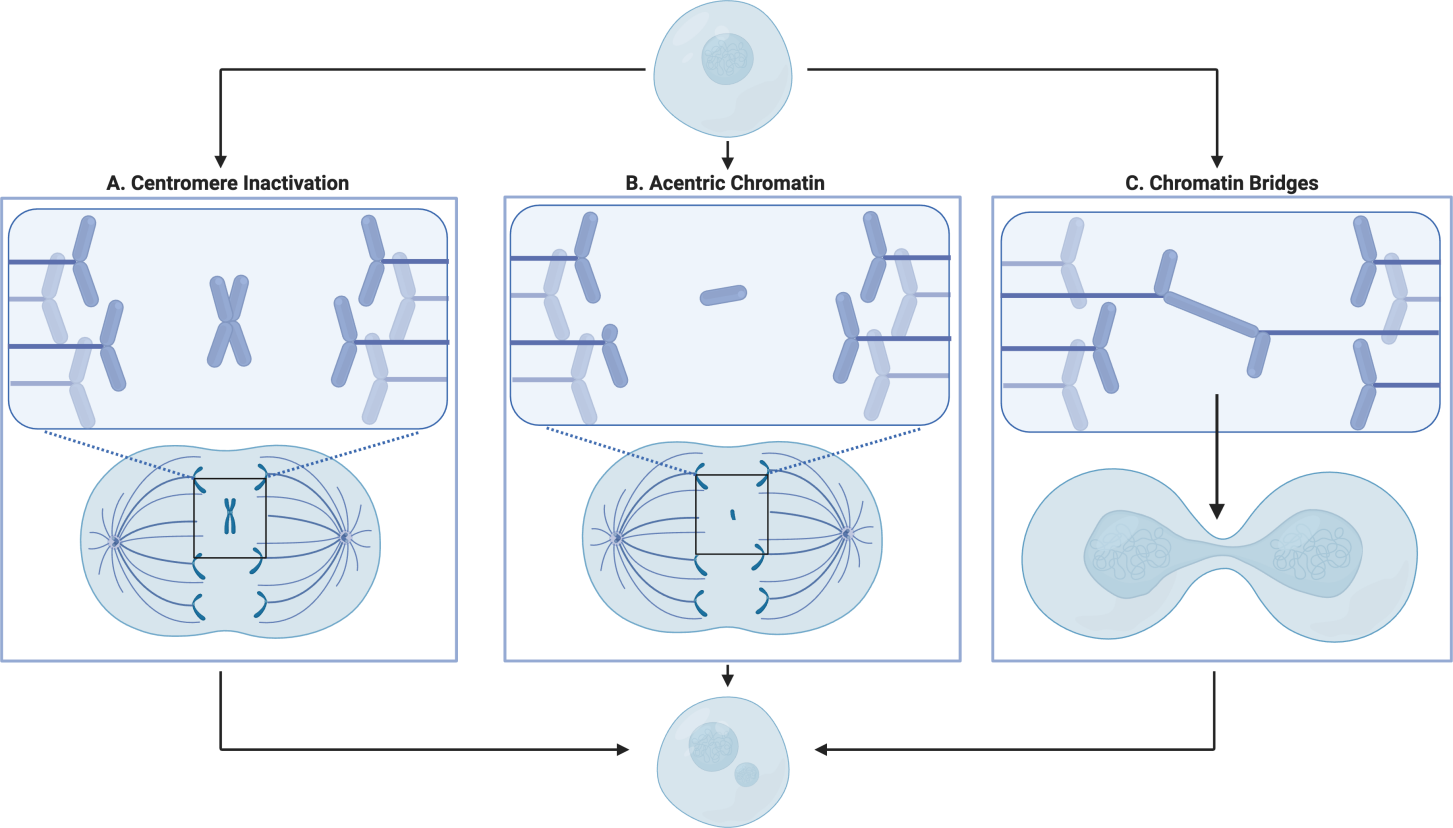
Mechanisms of MN formation. Cells generate MN through a number of different means. The failure of chromosomes to interact with the spindle apparatus may lead to the ‘abandonment’ of chromatin at the metaphase plate. This failure to properly segregate can occur due to centromere inactivation (A) and loss of the centromere (B). During segregation, lagging chromosomes/chromatin may form their own nuclear envelope generating a separate miniature nuclear body from the primary nucleus. Dicentric chromosomes pulled apart during mitosis may generate chromatin bridges during division. Breakage of these bridges generate fragments of chromatin that may progress into MN during nuclear envelope reformation (C)*.*

Cancer arises from aberrations in somatic cells which confer uncontrolled division that compromises the health of the host. This review highlights the process of chromothripsis downstream of micronuclei (MN) formation as a driver contributing to these abnormalities. First characterized more than 100 years ago, by cytologists Henry Howell and Justin Marie Jolly, MN have historically existed in the backdrop of cancer biology as a histological tool to quantify genotoxic stress [1]. MN can be found in several contexts in increasing frequency with age, neurodegenerative diseases, viral infections and of course, cancer [2–5]. Despite their pervasive association with pathology, MN remain a relatively unexplored avenue of research. Studies investigating the link between MN, cancer risk, metastasis and prognosis highlight MN’s role in contributing to cancer progression [6–8]. MN are a variety of chromosomal instability, a hallmark of cancer that promotes the acquisition of traits that support cancer survival and development [9]. MN can arise through several mechanisms. Perhaps the most well characterized model of MN formation suggests that they arise from lagging chromatin that fails to coordinate with the spindle apparatus during anaphase. Failure to recruit the spindle apparatus can occur due to centromere inactivation (figure 1A) or when chromatin lacks a centromere, as seen in acentric chromatin fragments (figure 1B) [10,11]. MN may also form from chromatin fragments generated from the breakage of chromatin bridges during division (figure 1C) [12,13]. Additionally, the S-phase generation of MN derived from extrachromosomal DNA has been documented but remains poorly understood [14,15]. For a more in-depth discussion on how MNs are generated, please refer to the review by Mazzagatti *et al.* [16].

Relative to the primary nucleus, MN exhibit attenuated recruitment of lamina and nuclear pore complex (NPC) components generating a nuclear environment permissive to rupture [17,18]. Loss of compartmentalization decouples MN chromatin from the information-preserving function of the nuclear membrane. This renders MN chromatin vulnerable to fragmentation due to interactions with the cytosol creating the potential for chromothripsis [19]. Chromothripsis describes the process by which a chromosome shatters and is re-ligated in random fashion generating a derivative chromosome [20]. Cancers exhibiting chromothriptic karyotypes are associated with a higher risk of metastasis, shorter survival and chemotherapy resistance [21,22]. Given this association, it is imperative to understand the link between MN and chromothripsis. This review will characterize how MN catalyse chromothripsis and the implications of chromothriptic chromosomes with regards to promoting malignancy.

## 2. How do micronuclei promote chromothripsis?

The dogma of cancer development has largely been dominated by the notion that cancer cells arise from the gradual accumulation of mutations over time [23]. The progression of colorectal cancer from adenoma to invasive carcinoma is illustrative of this gradualistic model. Comparative analysis of mutations between adenomas and carcinomas reveals a stepwise progression initiated by a loss of tumour suppressor APC function, followed by mutations dysregulating the oncogene RAS, and finally the loss of tumour suppressor function p53 with the transition from advanced adenoma to carcinoma [24,25]. While the gradual accumulation of mutations may explain some forms of cancer progression, this model fails to capture the full spectrum of carcinogenesis. Genomic analysis of primary and metastatic ductal adenocarcinoma, for example, reveals that approximately 65% of tumours exhibit complex chromosomal rearrangements spanning a single or few mitotic events [26]. In a subset of tumours screened, these rearrangements corresponded to the simultaneous loss of functionality in multiple tumour suppressor genes [26]. This rapid acquisition of malignancy highlights how carcinogenesis can occur in an ‘all at once’ event as opposed to the sequential accumulation of mutations. Facilitating these events are complex chromosomal rearrangements which fall under three primary classifications: chromothripsis, chromoanasynthesis and chromoplexy. The most well understood of the three being chromothripsis [27]. For further information on chromoanasynthesis and chromoplexy see the reviews by Pellestor & Gatinois [28] and Shen [29]. Pertinent to the discussion of MN, chromatin sequestered within MN exhibits progressive fragmentation and rearrangement characteristic of chromothripsis [30]. The precise mechanism underlying this association is not fully understood although several models have been put forward ranging from nuclease-mediated digestion of chromatin following rupture, condensation-dependent replication fork collapse, micro homologous annealing between stalled replication forks and/or base excision repair of R-loops.

### 2.1. Nuclear envelope defects preceding rupture

Rupture of the MN nuclear envelope is significantly associated with MN DNA damage making it a promising starting point for understanding MN chromothripsis [19,31]. MN membranes are thought to be prone to rupture due to the altered composition of their nuclear envelopes relative to the primary nucleus. The nuclear envelope is a double-layered lipid bilayer matrix invaginated by the NPC and structurally reinforced by a network of intermediate filaments known as the nuclear lamina [31]. In contrast to primary nuclei, MN exhibit defects in terms of their ability to recruit both components of the NPC and lamin B [18,31]. This defective recruitment may be at least partially the result of chromatin positioning during telophase. Profiling of MN derived from lagging chromatin reveals that positioning within the spindle midzone during telophase correlated inversely with recruitment of both lamin B and the NPC [19]. In contrast, lagging chromosomes positioned towards the periphery went on to form MN rescued not only in terms of lamin B/NPC recruitment but also exhibiting significantly less DNA damage relative to their spindle midzone counterparts [19].

In addition to chromatin positioning during telophase, the recruitment of nuclear envelope components to MN appears partially dependent on physical characteristics of the MN. The efficiency of lamin B recruitment decreases as nuclear membrane curvature increases [32]. This results in reduced lamin B deposition at smaller MN [17]. Likewise, the lack of structural reinforcement due to lamin B’s absence makes smaller MN more prone to rupture [33]. Accordingly, the size of the MN predicts not only lamin B recruitment but also the probability of rupture [33]. Unlike lamin B, lamin isoforms A/C incorporate within the MN nuclear envelope at similar levels to the primary nucleus [34]. This does not necessarily mean lamin A/C is not implicated in rupture, however. The rupture of lamin B negative MN appears partially dependent on the phosphorylation of lamin A/C Ser 395 by the kinase ATR. Inhibition of ATR diminishes the population of ruptured MN by approximately half in lamin B1 negative MN while having no effect on rupture in lamin B1 positive MN [35,36]. This suggests that lamin A/C plays a role in compensating for maintaining nuclear membrane integrity in lamin B1 negative MN [35,36].

### 2.2. Nuclease invasion following nuclear envelope rupture

**Figure 2.**
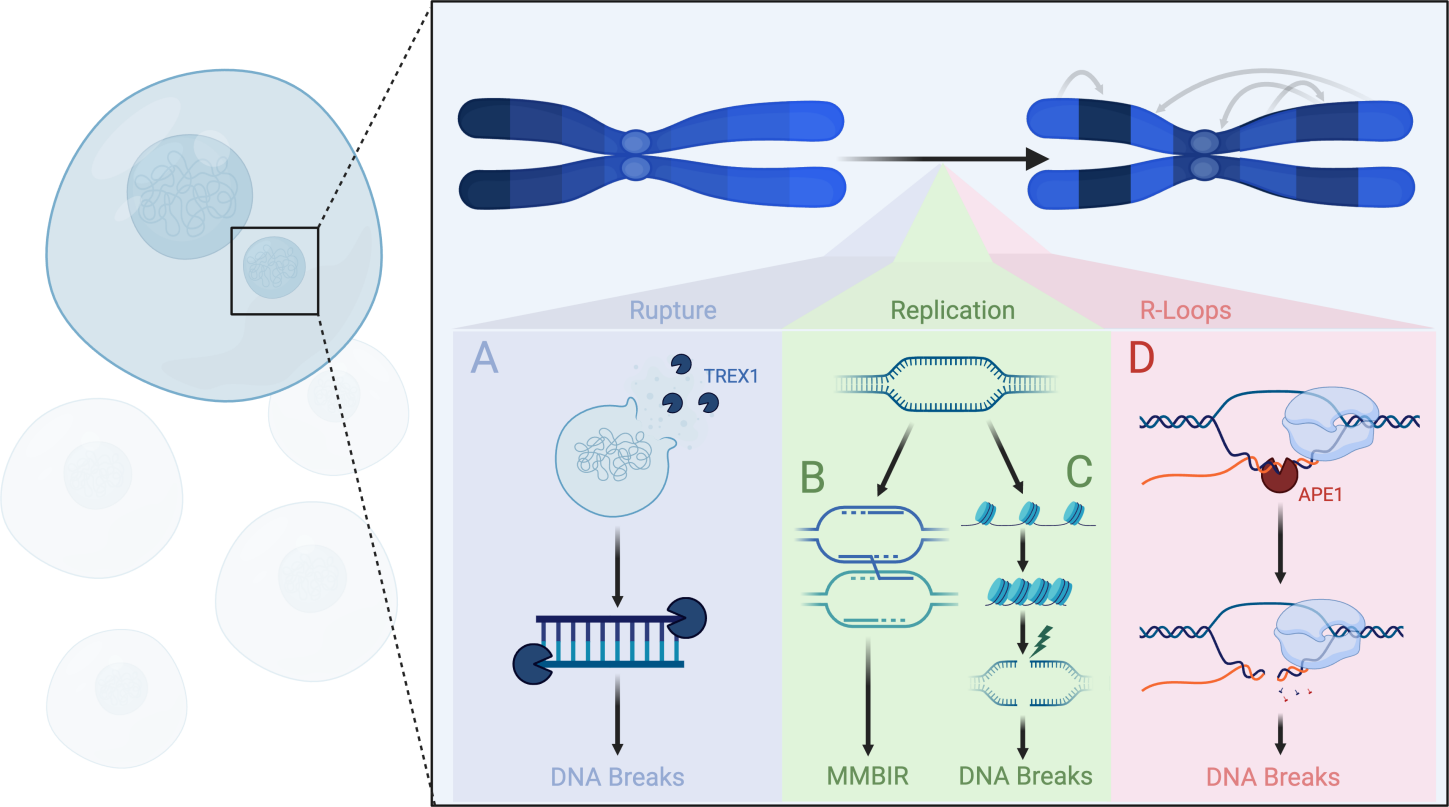
Mechanisms of chromothripsis. MN putatively promote chromothripsis through a number of proposed models. Invasion of MN by cytoplasmic nucleases such as TREX1 may catalyse the formation of DNA breaks which progress into complex chromosomal rearrangements characteristic of chromothripsis (A). Alternatively, defective import of replication factors into the MN may promote the stalling of replication forks and subsequent hijacking of active forks by their stalled counterparts. This hijacking may promote chromosomal rearrangements characteristic of chromothripsis (B). Similarly, replication forks stalling may subject DNA to mechanically generated lesions downstream of tensile forces generated by chromatin condensation following exit from S phase. The resulting chromosomal fragments, in turn, may result in the formation of chromothriptic chromosomes (C). MN chromothripsis may also be the result of R-loop formation downstream of retroelement dysregulation and/or defective import of transcriptional machinery. Subsequent R-loops may be subject to base excision repair and the generation of single-strand breaks (SSB) by the endonuclease APE1. If unresolved, these breakage sites may drive the fragmentation of chromatin and chromothripsis (D).

The rupture of MN due to lamin deficiency may be exacerbated by defects in nuclear envelope repair. The ESCRT-3 complex, which reseals lesions in the nuclear membrane, appears to be inhibited from the MN due to accumulation of the autophagic receptor p62 [37]. Over accumulation of ESCRT-3 at MN has also been associated with rupture suggesting either hypo-accumulation or hyper-accumulation to potentially presuppose rupture [38]. In 40–70% of ruptured MN, the exonuclease TREX1 is recruited [39]. Canonically, TREX1 mediates the degradation of cytosolic DNA [40,41]. The induction of MN rupture may drive TREX1 to recognize MN as reservoirs of cytosolic DNA facilitating their invasion (figure 2A) [31,39]. In line with this function, TREX1 KO correlates to a loss of DNA double-strand break-associated (DSB) histone variant γH2AX from ruptured MN [39]. TREX1’s role with regards to generating DSB within ruptured MN makes it an attractive candidate for MN chromothripsis. Indeed, TREX1 has been suggested to play a key role in mediating chromothripsis during cellular processes such as telomere crisis [42]. It is important, however, to note that there currently exists a gap in the literature demonstrating that TREX1-mediated DSB progresses into chromothripsis within the context of the MN. Altogether, these findings suggest that defects in the MN nuclear envelope predispose MN to rupture. TREX1, recruited to ruptured MN, subjects MN DNA to degradation potentially facilitating chromothripsis.

### 2.3. Replication fork delay

Depletion of the nuclear lamina may also mediate MN chromothripsis by driving replication fork delay. Replication forks are structures formed during DNA replication when double-stranded DNA bifurcates to reveal two single-stranded DNA templates. During cellular division, these replication forks traverse the entire human genome to copy the original DNA. Even in healthy cells, the progression of replication forks is frequently stalled upon encountering DNA lesions, non-canonical DNA structures and DNA associated transcriptional machinery [43–45]. The resolution of these delayed forks appears partially dependent upon the recruitment of proteins RPA, RAD51 and FANCD2 by the nuclear lamina [46–48]. Upon delay, filamentous actin propagates at sites of fork stress and actively repositions delayed forks towards the nuclear lamina through a myosin-based transport system. At the nuclear periphery, RPA, RAD51 and FANCD2 resolve delayed forks allowing for replication fork restarting [47,49]. In line with this model, lamina-deficient MN exhibits both defective recruitment of fork resolving factors and replication fork delay relative to the primary nucleus [19,50,51].

### 2.4. Premature chromatin condensation and microhomology-mediated break-induced replication

Replication fork delay presupposes two potentially non-mutually exclusive hypotheses for MN chromothripsis. In one hypothesis, replication fork delay causes the MN to progress through the cell cycle at a slower rate than the primary nucleus. While the primary nucleus enters prophase and subsequently condenses its chromatin, the MN undergoes this condensation with replication forks still active causing catastrophic pulverization of chromatin (figure 2C) [52–54]. In support of this model, premature induction of chromosomal condensation through the addition of calyculin A promotes fragmentation of MN chromatin for cells in G2 but not in G1 [10]. This finding suggests MN chromatin fragmentation is dependent on entry into the S phase putatively implicating the presence of replication forks as a prerequisite for chromothripsis. Correspondingly, MN generated in nocodazole-synchronized cells enter the cell cycle with minimal DNA damage but exhibit a dramatic increase in γH2AX staining upon entry into S phase [51].

Hypothesis two proposes that delayed replication forks invade adjacent replication forks through microhomology-mediated break-induced replication (MMBIR) generating complex chromosomal rearrangements characteristic of chromothripsis. (figure 2B) [55,56]. Substantiating this framework, single cell sequencing of MN chromothriptic chromosomes yields microhomology at more than 50% of breakpoint junctions [51]. This non-random annealing suggests that chromothriptic breaks are resolved in a manner that favours spatial proximity as would be expected under adjacent fork-dependent DNA repair [57]. Taken together these findings indicate that lamina depletion in MN inhibits their ability to recruit factors involved in resolving stalled replication forks. The accumulation of these stalled forks in MN may catalyse chromothripsis by causing MN chromatin to condense with replication forks still open causing catastrophic pulverization of chromatin. Alternatively, stalled replication forks may invade adjacent replication forks driving complex chromosomal rearrangements characteristic of chromothripsis.

### 2.5. Enrichment of R-loops in micronuclei

Other putative mechanisms of MN chromothripsis implicate the formation of RNA–DNA hybrids/R-loops (RL) as a mediator of chromatin fragmentation. Molecular analysis of MN reveals enrichment in RL however, the basis for this enrichment is not fully understood [58]. Endogenous retroelements are mobile genetic elements capable of auto-replication and insertion within the host genome. Canonically, healthy cells sequester these elements within heterochromatin impeding their spread [59]. Epigenetic reprogramming accompanying carcinogenesis, however, has the prospect of reversing this dormancy [60]. Correspondingly, the expression of retroelements has been observed to increase in cases of melanoma, breast, prostate and cervical cancer [61]. Deregulation of these elements may create an environment permissive to both the formation of RL and MN hence their association [62,63]. Alternatively, RL enrichment within MN could be the result of their aberrant proteome. MN characteristically exhibit defective recruitment of transcriptional elongation factors CDK9, CDK12, the spliceosome components SRSF1/SF3B1 and RNA pol 2 [5,64]. This depletion of transcriptional machinery may hinder transcriptional progression potentially facilitating the invasion of the DNA duplex by nascent RNA forming RL [65]. RL, in turn, may initiate the base excision repair pathway through the coordinated recruitment of enzymes ADAR and MPG. The resulting abasic site acts putatively as a substrate for the formation of SSB through the action of the endonuclease APE1 (figure 2D) [58,66]. The attenuated recruitment of DNA repair factors characteristic of MN may prevent the resolution of SSB allowing them to progress into DSB in the event of DNA replication or corresponding SSB on complementary strands of the DNA duplex.

Whether MN chromothripsis occurs as the downstream product of TREX1, premature chromatin condensation, MMBIR, RL or a combination of the four remains an area of open debate. Regardless of the model, MN appear to promote large-scale chromosomal rearrangements characteristic of chromothripsis [51]. The induction of chromothripsis crucially allows cancer cells to circumvent the limitations of clonal division and generate progeny with the potential of acquiring traits that permit clonal expansion.

### 2.6. Chromothriptic reintegration

The rejoining of MN chromosomes with the primary nucleus creates a mechanism by which chromothripsis-driven alterations can assimilate into the cancer genome [10,30,67,68]. Reintegration of MN chromosomes into the primary nuclear body occurs in approximately 10–40% of MN bearing cells following entry into mitosis [69,70]. Despite the frequency of reintegration, MN derived chromosomes exhibit several defects in terms of their ability to recruit factors required for mitosis [71]. Fluorescent staining of chromosomes derived from MN re-entering mitosis reveal depleted recruitment of the mitotic checkpoint component MAD1 in addition to Aurora B, a kinase responsible for facilitating connections between mitotic spindles and the kinetochore [71]. Similarly, MN chromosomes exhibit a significant reduction in the recruitment of the centromere defining histone variant CENP-A, kinetochore components CENP-C/T and the kinetochore-associated epigenetic modification H4K20me1 [71]. The reductions in these components may be due to MN import defects. The CENP-A-associated chaperone HJURP, H4K20me1 writer KMT5A (SETD8), CENP-C and CENP-T are all transported across the NPC of which MN frequently lack [71]. Given these deficiencies, how MN derived chromatids participate in mitosis while exhibiting kinetochore defects remains an open question worthy of investigation. In addition to defective kinetochore factor recruitment, MN-derived chromosomes bearing chromothriptic signatures often exist as ‘shattered’ chromatin fragments of their original chromosome outright lacking a functional centromere [11,30]. How these acentric fragments faithfully partition into daughter cells remains unclear.

**Figure 3.**
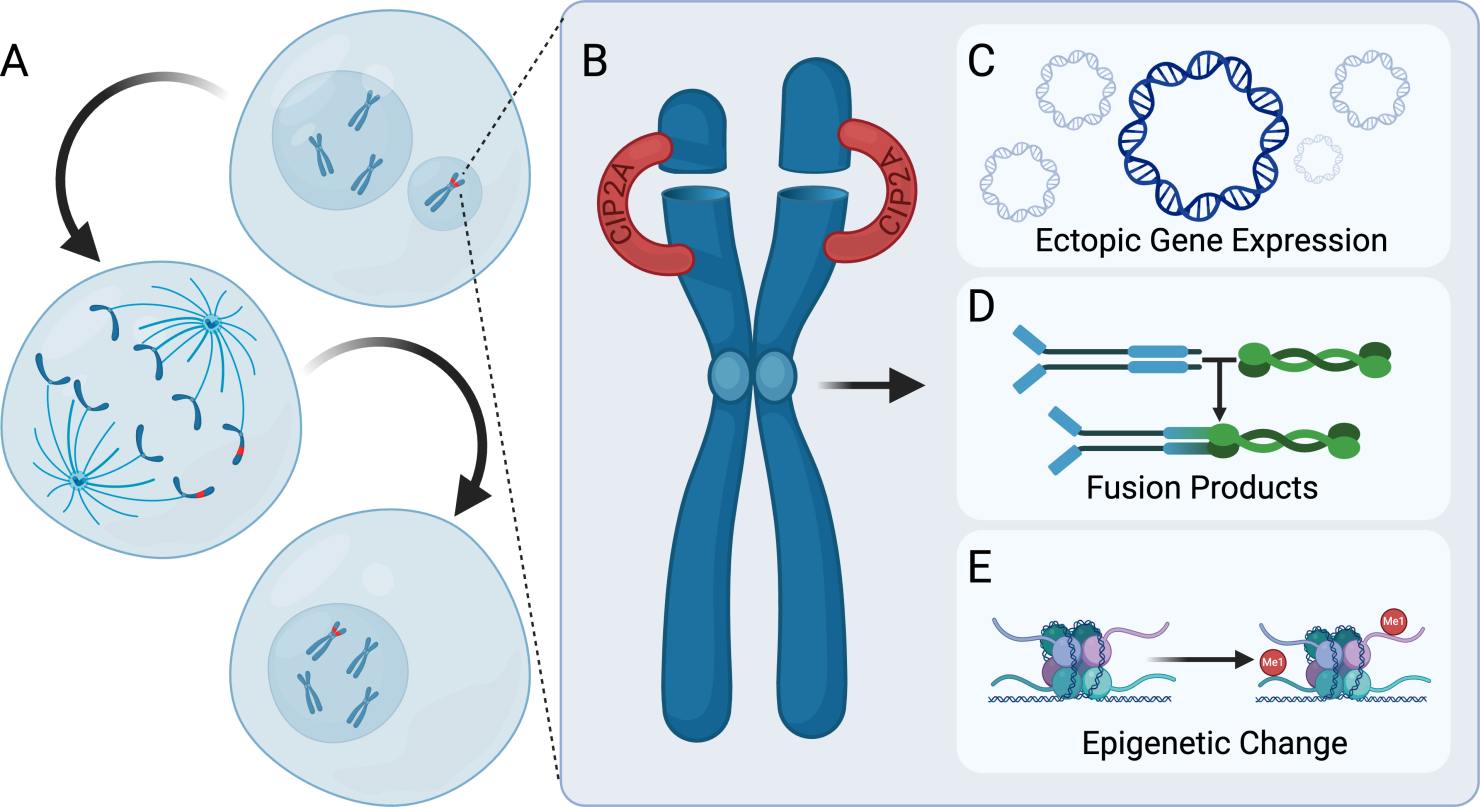
Consequences of chromothripsis and reincorporation of chromothriptic chromosomes. Chromosomes undergoing chromothripsis have the potential of rejoining the primary nucleus during subsequent mitosis (A). This reintegration process may be facilitated through the action of protein complex CIP2A-TOPB1 which promotes the clustering of chromothriptic fragments allowing for faithful entry into mitosis (B). Chromothriptic chromosomes, in turn, may catalyse a number of events that promote the survival of cancer. Specifically, chromothripsis may promote the silencing of tumour suppressors and amplification of oncogenes (C), the formation of fusion products (D) and epigenetic reprogramming (E)*.*

One particularly promising model posits this reintegration to be the result of fragment clustering. Facilitating this clustering is the localization of protein complex CIP2A-TOPB1 to sites of DSB (figure 3A,B) [72]. This localization is potentially mediated through direct recruitment by the DNA damage response protein MDC1 which respectively associates with γH2AX positive DNA lesions. SiRNA-based depletion of either CIP2A, TOPB1 or MDC1 was associated with random distribution of chromatin fragments following mitosis. Moreover, depletion of CIP2A negatively impacted cancer cell viability while having no significant impact on untransformed cells [73,74]. With CIP2A expression being nonessential to healthy cells, this finding frames CIP2A as a potentially viable therapeutic target for selectively eliminating chromosomally unstable cells.

### 2.7. Micronuclear epigenetic defects

Moreover, epigenetic signatures, transcriptional defects and differential chromatin accessibility acquired in the MN appears to persist through reintegration causing enduring changes to the primary nuclear epigenome (figure 3E) [69,75]. Experimental investigation of this phenomenon reveals MN chromatin to be depleted of histone acetylation marks (H3K9ac, H3K27ac and H3K14ac), enriched in histone methylation marks (H3K9me3, H3K27me3 and H3K36me3) and depleted of histone ubiquitin marks (H2AK119ub and H2BK120ub) relative to the primary nucleus [75]. MN bearing these ectopically deposited epigenetic marks were strongly associated with reduced transcription [69,75]. In particular, the loss of the active marks H3K27ac and H3K9ac corresponded with a reduction in the ability of MN to recruit RNA polymerase 2 [69,75]. Curiously, despite the reported increase in terms of repressive heterochromatin associated marks, ATAC-seq performed on MN reveals that MN chromatin is substantially biased towards having increased accessibility at gene bodies and their respective promoters while exhibiting decreased accessibility at intronic/intergenic regions [69,75]. Critically, MN-derived chromatin maintains its epigenetic signature, transcriptional defects and differential chromatin accessibility upon reincorporation into the primary nucleus [69,75]. MN chromatin may therefore serve an additional function in acting as a disruptor of epigenetic homeostasis following reintegration. More work, however, is necessary to elucidate the long-term consequences of this reintegration with regards to cell fate.

## 3. Chromothripsis as a driver of malignancy

A cell unimpeded in division is expected to double at an exponential rate. Investigation into the initial stages of tumour development is consistent with this model [76]. The exponential model, however, fails to account for the tendency of tumours to decrease in growth rate later in progression [77]. This limit on cancer cell growth is imposed through a combination of finite resources, the build up of toxic metabolites, immune targeting, chemotherapy and radiotherapy among other factors [78–81]. In conjunction, these drivers provide a selective pressure for the acquisition of phenotypes which oppose these limits on division. These so-called cancer ‘hallmarks’ constitute traits that increase the fitness and clonal expansion of a cancer cell population [9]. Fluctuations in gene copy number characteristic of chromosomal instability (CIN) may catalyse the acquisition of these hallmarks [82]. Consequently, CIN is associated with immunosuppression, metastasis and an overall poor prognosis [83,84]. The process of chromothripsis constitutes one form of CIN. Cytogenetic profiling of MN chromosomes reveals a 120-fold higher rate of translocations, insertions and deletions as a byproduct of chromothripsis [85]. Most of these rearrangements are either neutral or deleterious to cancer cells. Under the right circumstances, however, these stochastic rearrangements confer a survival advantage through tumour suppressor inactivation, oncogene amplification and generation of fusion products.

### 3.1. Genetic alterations downstream of chromothripsis: tumour suppressor silencing

The clustered rearrangements characteristic of chromothripsis are liable to generate derivative chromosomes carrying both copy number gains and losses [21]. Pertinent to the discussion of tumour suppressors is this capacity to drive copy number loss. Tumour suppressors are genes that regulate cellular division. Loss of tumour suppressor function correspondingly drives unchecked cellular proliferation increasing the risk of a cell undergoing neoplastic transformation [86]. Perhaps the most well-characterized tumour suppressor is p53, a transcription factor that acts as an interface between stress signalling and cell cycle progression (table 1) [115]. Associations between chromothripsis and deactivation of the tumour suppressor gene p53 have been documented in acute myeloid leukaemia, medulloblastomas, osteosarcomas and an array of paediatric cancers [116–118]. In breast cancer, the loss of p53 function due to copy number alteration driven by chromothripsis of the p53 locus on chromosome 17 is particularly overrepresented [109]. In addition to p53, genomic analysis of osteosarcomas has revealed copy number alterations of tumour suppressors RB1, WWOX, DLG2 and LSAMP to correspond to their enrichment at sites that exhibited complex breakage patterning characteristic of chromothripsis (table 1) [94]. Similarly, genomic profiling of colorectal cancers has found the localization of tumour suppressors FH, MLL3 and EXO1 to sites exhibiting chromothriptic patterning corresponded to structural rearrangements which silenced gene functionality (table 1) [101].

**Table 1.** A summary of genes altered due to chromothripsis. Gene abbreviations used: CCND1, cyclin D1; CDK4/6, cyclin-dependent kinase 4/6; DLG2, discs large MAGUK scaffold protein 2; EGFR, epidermal growth factor receptor; ERB2/3, erb-b2 receptor tyrosine kinase 2/3; EXO1, exonuclease 1; FH, fumarate hydratase; HMGA2, high-mobility group AT-hook 2; LSAMP, limbic system-associated membrane protein; MDM1/2, mouse double minute 1/2; MLL3, mixed-lineage leukaemia protein 3; MYC, myelocytomatosis oncogene protein; P53, tumour protein 53; RB1, retinoblastoma protein 1; SETDB1, SET domain bifurcated 1; SOX2, SRY-box transcription factor 2; TERT, telomerase reverse transcriptase; WWOX, WW domain-containing oxidoreductase.

gene	class	function	chromothriptic alteration	cancer t**ype**
CCND1	oncogene	coordinates with CDK4 to drive cell cycle progression [87]	copy number gain [88]	multiple [88]
CDK4/6	oncogene	phosphorylates cell cycle machinery allowing for cell cycle progression [89]	copy number gain [88], extrachromosomal amplification [90–92]	multiple [88,90–92]
DLG2	tumour suppressor	regulator of DNA repair and cell cycle progression [93]	copy number loss [94]	osteosarcoma [94]
DYRK1A	oncogene	kinase regulating the activation of targets involved in cell cycle progression, splicing, stemness, the DNA damage response and transcription [95]	copy number gain [96]	myoproliferative neoplasm [96]
EGFR	oncogene	receptor tyrosine kinase regulating cellular proliferation [97]	copy number gain [98], extrachromosomal amplification [90–92]	glioblastoma [98] multiple [90–92]
ERBB2/3	oncogene	receptor tyrosine kinase regulating cellular proliferation [99]	copy number gain [88], extrachromosomal amplification [90–92]	multiple [88,90–92]
EXO1	tumour suppressor	exonuclease mediating the excision of strands carrying mis-incorporated based during DNA mismatch repair [100]	gene silencing breakpoint [101]	colorectal cancer [101]
FH	tumour suppressor	component of the TCA cycle that supports mitochondrial function and maintains prolyl hydroxylase activity preventing hypoxic signalling [102]	gene silencing breakpoint [101]	colorectal cancer [101]
HMGA2	oncogene	transcription factor promoting expression of proteins implicated in the epithelial to mesenchymal transition [103]	extrachromosomal amplification [90–92]	multiple [90–92]
LSAMP	tumour suppressor	regulator of epithelial to mesenchymal transition [104]	copy number loss [94]	osteosarcoma [94]
MDM1/2	oncogene	inhibits the activity of tumour suppressor P53 [105]	copy number gain [88], extrachromosomal amplification [90–92]	multiple [88,90–92]
MLL3	tumour suppressor	histone methyltransferase regulating the expression of tumour suppressor genes [106]	gene silencing breakpoint [101]	colorectal cancer [101]
MYC	oncogene	transcription factor regulating DNA repair pathways, cellular proliferation, apoptotic signalling, differentiation, immune response and metabolism [107]	copy number gain [88], extrachromosomal amplification [90–92]	multiple [88,90–92]
P53	tumour suppressor	modulates cell cycle progression and initiates apoptosis in the presence of cellular stress signalling [108]	copy number loss [109]	breast cancer [109]
RB1	tumour suppressor	regulates the transition from G1 to S phase through inhibition of S phase mediating transcription factor E2F [110]	copy number loss [94]	osteosarcoma [94]
SETDB1	oncogene	chromatin modifying element regulating activation of tumour suppressor genes [111]	copy number gain [88]	multiple [88]
SOX2	oncogene	transcription factor that is responsible for activating the self-renewing capacity of stem cells [112]	extrachromosomal amplification [90–92]	multiple [90–92]
TERT	oncogene	component of the telomerase complex allows for the immortalization of cancer cells [113]	extrachromosomal amplification [90–92]	multiple [90–92]
WWOX	tumour suppressor	mediator of proapoptotic signalling in the presence of DNA damage [114]	copy number loss [94]	osteosarcoma [94]

### 3.2. Genetic alterations downstream of chromothripsis: activation of oncogenes

While the loss of tumour suppressor functionality is an integral feature of tumorigenesis, it is equally important to consider the role of oncogenes in promoting neoplastic expansion. Oncogenes are distinguished from tumour suppressors in that their activation confers cell cycle progression [86]. The kinase DYRK1A, for example, is an oncogene that mediates the activation of genes involved in proliferation, DNA repair, differentiation and cellular survival (table 1) [96]. Ectopic activation of DYRK1A appears to play a role in the progression of myoproliferative neoplasms to blast phase; a transition that corresponds to a dismal prognosis with a median overall survival of three to five months [119]. Multi-omics profiling of patients exhibiting this leukaemic transformation reveals a pervasive pattern of chromothripsis affecting chromosome 21. This patterning was associated with copy number gain in the locus corresponding to the gene DYRK1A [96]. This suggests chromothripsis plays a role in activating oncogenes implicated in the blast phase transition. The chromothriptic-dependent activation of oncogenes appears to be a motif present across several cancer types. Genomic sequencing of glioblastoma reveals a link between the copy number gain of oncogenes EGFR and MDM1/2 and their positioning in regions exhibiting clustered rearrangements consistent with chromothripsis (table 1) [98]. Moreover, whole genome sequencing across 2658 tumours and 38 cancer types reveals copy number gain of oncogenes CCND1, CDK4, MDM2, SETDB1, ERBB2/3 and MYC to correspond to their respective gene loci exhibiting chromothriptic patterning (table 1) [88].

### 3.3. Genetic alterations downstream of chromothripsis: extrachromosomal DNA generation

Intrachromosomal copy number gain, however, only constitutes one facet of chromothriptic oncogene amplification. In addition to generating complex chromosomal rearrangements, chromothripsis is implicated in the formation of circularized extrachromosomal DNA fragments (figure 3C). Chromothripsis is thought to generate extrachromosomal DNA through the NHEJ-dependent circularization of excised chromatin fragments [120,121]. This relationship is perhaps best illustrated by the characterization of cell line SCLC-21H undergoing chromothripsis at chromosome 8 [121,122]. Curiously, while chromosome 8 oscillated between low copy number states, discrete sequences of the chromosome presented with copy numbers ranging from 50 to 200 [122]. FISH staining of this highly amplified sequence revealed it to be distinctly absent from the chromothriptic chromosome 8. Rather, probes aligned to this highly amplified sequence-stained fragments of extrachromosomal DNA. Contained within this sequence was the oncogene MYC (table 1) [122]. Altogether, these findings suggest that following chromothripsis of chromosome 8, the locus containing MYC was excised into an extrachromosomal fragment which underwent a series of amplification events. Extrachromosomal DNA replicates concordant with chromosomal DNA but notably is partitioned randomly upon division [123]. The unequal distribution of extrachromosomal DNA during mitosis means that genes contained within have the potential to reach copy numbers higher relative to intrachromosomal means of amplification [124]. These extrachromosomal amplification events are thought to be relatively common across cancer types. Analysis of data sourced from The Cancer Genome Atlas and The Pan-Cancer Analysis of Whole Genomes reveal extrachromosomal DNA to be present in 14.3% of cancer samples screened [90]. Genes contained within extrachromosomal DNA were also non-random in terms of sequence with 53.5% containing a form of oncogene such as EGFR, CDK6, CDK4, ERBB2, MYC, MDM2, HMGA2, SOX2 and TERT among others (table 1) [90–92].

### 3.4. Genetic alterations downstream of chromothripsis: fusion products

Beyond amplification and silencing of genes, chromothripsis also functions as a means for cancer cells to generate oncogenic fusion products (figure 3D). Fusion products are the result of DNA rearrangements which combine genes to generate a chimeric RNA/protein with altered function. In the case where these rearrangements involve genes implicated in regulating proliferation or cellular survival, oncogenic fusion products may arise [125]. The presence of specific fusion products varies largely by cancer subtype and tumour grade allowing for their use as a diagnostic/prognostic marker [125]. Supratentorial ependymomas, for example, are a tumour of the CNS characterized by the presence of the chimeric protein ZFTA::RELA [126]. RELA is a subunit of the transcription factor complex NF-κB which activates genes implicated in cellular proliferation, metastasis and anti-apoptotic pathways [127]. The precise function of ZFTA is currently unknown, however, its association with RELA drives constitutive localization of RELA to the nucleus allowing for ectopic activation of NF-κB signalling [128,129]. Genomic analysis of supratentorial ependymoma tumours reveals oscillating copy number states characteristic of chromothripsis to be a common feature of chromosome 11q [130–132]. Regions of the 11q chromosome exhibiting chromothriptic patterning differed by tumour sample with the notable exception of a shared region of the 11q chromosome fusing the genes ZFTA and RELA [130]. These findings indicate chromothripsis plays a significant role in mediating the generation of fusion products which drive supratentorial ependymoma carcinogenesis.

Beyond supratentorial ependymomas, chromothripsis derived oncogenic fusion products have been documented with regards to PVT1::MYC in medulloblastoma, RLF::MYCL1 in small cell lung cancer and HNRNPM::LEUTX in small round cell neoplasms [133–136]. Many chromothripsis derived fusion products, however, remain uncharacterized in terms of oncogenic potential. The generation of fusion products NDUFAF2::MAST4 in prostate cancer and PVT1::NDRG1 in medulloblastomas are both associated with chromothripsis but remain poorly-defined in terms of contribution to malignancy [135,137]. Overall, chromothripsis contributes to the complex landscape of genetic alterations observed in cancers not only by silencing tumour suppressors and amplifying oncogenes but also by generating fusion proteins that can contribute directly to malignancy.

## 4. Conclusion and future perspective

The aim of this review has been to highlight MN as an underexplored therapeutic target by characterizing the link between MN and malignancy. Altogether this association stems from the presence of a defective proteome within MN rendering its chromatin liable to fragmentation due to interactions with cytosolic nucleases, stalled replication forks and RL. The stochastic rejoining of these fragments generates derivative chromosomes bearing dysfunctional or absent tumour suppressors, overexpressed oncogenes and chimeric gene products with altered function. Chromothriptic chromosomes bearing these mutations can reintegrate with the primary nuclear genome allowing for the faithful propagation of genetic and epigenetic modifications derived from the MN across cellular division.

In total, these modifications contribute to genetic change that fuels clonal expansions conferring decreased survival time and increased tumour aggressiveness [138,139]. Correspondingly, MN have been shown to be a valuable predictive measure in appraising the malignant potential of cervical, colorectal and breast cancers [139–141]. Beyond this role as a diagnostic tool, however, MN remain untapped in terms of fulfilling the role of an actual therapeutic target.

This is not to say that such a goal should remain unrealized. Looking into the future, MN are highly attractive targets for therapies that leverage synthetic lethality. Synthetic lethality refers to an interaction where the combination of two events leads to cell death. This principle is well illustrated in the case of PARP inhibition as a mechanism of treatment for BRCA1/2 deficient cancers [142]. PARP acts to prevent SSB from progressing into DSB while BRCA1/2 resolves DSB through homologous recombination. Inhibition of PARP is lethal in the BRCA incompetent cancer cells while being well tolerated by normal cells [142]. The strategy of synthetic lethality could be analogously implemented to target chromosomally unstable cells bearing MN. One promising approach is to target the kinesin KIF18A. KIF18A functions to promote chromosome congression during mitosis [143]. While this congression is disposable to normal cells, cells exhibiting chromosomal instability depend upon the action of KIF18A for division [144]. Correspondingly, depletion of KIF18A has been reported to selectively eliminate chromosomally unstable cells while having little effect on normal cells [145]. Alternative findings suggest that KIF18A may function as an intermediary in mediating the tumorigenic effects of MN [146]. Mice bearing MN induced by a null p53 mutation exhibit a marked increase in both genomic instability and spontaneous tumorigenesis [146]. In contrast, KIF18A null mice paradoxically exhibited similar MN loads to the p53 null group while maintaining comparable levels of genomic stability to wild-type mice and no increase in tumorigenesis [146]. Inactivation of KIF18A diminishes the tumorigenic potential of MN, highlighting its role as a link between MN and cancer progression [146]. KIF18A inhibition, of course, is just one of many possible therapeutic strategies that could leverage MN as a target of treatment. Anti-MN-based treatments are particularly promising in that they would be highly selective in targeting a subset of the cancer cell population highly predisposed to chromothripsis [19,30]. As such, targeting MN bearing cells could mean potentially diminishing cancer’s capacity to develop mutations that drive cancer progression. In this regard, MN, the century-old discovery of Dr Howell and Dr Jolly could represent the next great frontier with respect to treating cancer.

## Data Availability

This article has no additional data.
